# SARS-CoV-2 T Cell Response in Severe and Fatal COVID-19 in Primary Antibody Deficiency Patients Without Specific Humoral Immunity

**DOI:** 10.3389/fimmu.2022.840126

**Published:** 2022-03-10

**Authors:** Sophie Steiner, Tatjana Schwarz, Victor M. Corman, Laura Gebert, Malte C. Kleinschmidt, Alexandra Wald, Sven Gläser, Jan M. Kruse, Daniel Zickler, Alexander Peric, Christian Meisel, Tim Meyer, Olga L. Staudacher, Kirsten Wittke, Claudia Kedor, Sandra Bauer, Nabeel Al Besher, Ulrich Kalus, Axel Pruß, Christian Drosten, Hans-Dieter Volk, Carmen Scheibenbogen, Leif G. Hanitsch

**Affiliations:** ^1^Institute of Medical Immunology, Charité—Universitätsmedizin Berlin, Corporate Member of Freie Universität Berlin and Humboldt Universität zu Berlin, Augustenburger Platz 1 and Berlin Institute of Health, Berlin, Germany; ^2^Institute of Virology, Charité—Universitätsmedizin Berlin, Corporate Member of Freie Universität Berlin and Humboldt-Universität zu Berlin, and German Centre for Infection Research, Associated Partner, Charitéplatz 1, Berlin, Germany; ^3^Berlin Institute of Health at Charité—Universitätsmedizin Berlin, Berlin, Germany; ^4^Department of Infectious Diseases and Respiratory Medicine, Charité—Universitätsmedizin Berlin, Corporate Member of Freie Universität Berlin, Humboldt-Universität zu Berlin, and Berlin Institute of Health, Berlin, Germany; ^5^Department of Pulmonary Medicine, University Hospital Leipzig, Leipzig, Germany; ^6^Department of Pulmonary Medicine and Infectious Diseases, Vivantes-Klinikum Neukölln, Berlin, Germany; ^7^Department of Nephrology and Medical Intensive Care, Charité-Universitätsmedizin Berlin, Freie Universität Berlin, Berlin Institute of Health, Humboldt-Universität zu Berlin, Berlin, Germany; ^8^Department of Pulmonary Medicine and Infectious Diseases, Vivantes-Klinikum Friedrichshain, Berlin, Germany; ^9^Department of Immunology, Labor Berlin GmbH, Berlin, Germany; ^10^Department of Pediatric Respiratory Medicine, Immunology and Critical Care Medicine, Charité—Universitätsmedizin Berlin, Corporate Member of Freie Universität Berlin, Humboldt-Universität zu Berlin, and Berlin Institute of Health, Berlin, Germany; ^11^Institute of Transfusion Medicine, Charité Universitätsmedizin Berlin, Berlin, Germany; ^12^Berlin Institute of Health at Charité—Universitätsmedizin Berlin, BIH Center for Regenerative Therapies, Charitéplatz 1, Berlin, Germany; ^13^Berlin Center for Advanced Therapies, Charité—Universitätsmedizin Berlin, Berlin, Germany

**Keywords:** primary immunodeficiencies (PID), primary antibody deficiency (PAD), coronavirus disease 2019 (COVID-19), severe acute respiratory syndrome coronavirus 2 (SARS-CoV-2), convalescent plasma (CP), type I interferons, innate immunity

## Abstract

Morbidity and mortality of COVID-19 is increased in patients with inborn errors of immunity (IEI). Age and comorbidities and also impaired type I interferon immunity were identified as relevant risk factors. In patients with primary antibody deficiency (PAD) and lack of specific humoral immune response to SARS-CoV-2, clinical disease outcome is very heterogeneous. Despite extensive clinical reports, underlying immunological mechanisms are poorly characterized and levels of T cellular and innate immunity in severe cases remain to be determined. In the present study, we report clinical and immunological findings of 5 PAD patients with severe and fatal COVID-19 and undetectable specific humoral immune response to SARS-CoV-2. Reactive T cells to SARS-CoV-2 spike (S) and nucleocapsid (NCAP) peptide pools were analyzed comparatively by flow cytometry in PAD patients, convalescents and naïve healthy individuals. All examined PAD patients developed a robust T cell response. The presence of polyfunctional cytokine producing activated CD4^+^ T cells indicates a memory-like phenotype. An analysis of innate immune response revealed elevated CD169 (SIGLEC1) expression on monocytes, a surrogate marker for type I interferon response, and presence of type I interferon autoantibodies was excluded. SARS-CoV-2 RNA was detectable in peripheral blood in three severe COVID-19 patients with PAD. Viral clearance in blood was observed after treatment with COVID-19 convalescent plasma/monoclonal antibody administration. However, prolonged mucosal viral shedding was observed in all patients (median 67 days) with maximum duration of 127 days. PAD patients without specific humoral SARS-CoV-2 immunity may suffer from severe or fatal COVID-19 despite robust T cell and normal innate immune response. Intensified monitoring for long persistence of SARS-CoV-2 viral shedding and (prophylactic) convalescent plasma/specific IgG as beneficial treatment option in severe cases with RNAemia should be considered in seronegative PAD patients.

## Introduction

Severity of COVID-19 is associated with increased age, male sex, and comorbidities, such as diabetes, arterial hypertension or pulmonary disease (1). In patients with inborn errors of immunity (IEI), morbidity and mortality of COVID-19 is increased (2–9). However, IEI form a very heterogeneous group of patients (10). With <700 COVID-19 cases in IEI patients reported worldwide (11), a higher risk for severe disease courses was confirmed to occur in older patients and in those with comorbidities (2). Identified immunological host factors include type I interferon (IFN) autoantibodies or disruption of type I IFN signaling, affecting innate immune response to SARS-CoV-2 (12, 13). These autoantibodies are found frequently in APS-1 (autoimmune polyendocrine syndrome) patients (14), however general frequency of type I IFN autoantibodies in PAD patients are currently unknown and other immunological mechanisms underlying the predisposition to severe disease courses remain to be determined.

With regard to humoral immunity, early clinical data from XLA patients suggested that lack of SARS-CoV-2 humoral immunity may be sufficiently compensated by innate and T cell immunity in order to prevent severe COVID-19 (2, 15–20). On the other hand, patients with Good’s syndrome, a thymoma-associated hypogammaglobulinemia with B and/or T cell deficiency and lack of specific humoral immune response, were reported to be at significantly increased risk for severe COVID-19 and a high case fatality rate (12). Observations in immunocompetent patients with mild COVID-19 disease, where SARS-CoV-2-specific antibodies are undetectable in 10–15% (21), further highlighted the relevance of non-antibody mediated immunity and levels of specific T cellular immunity were found to be similar, regardless of the presence or absence of SARS-CoV-2 antibodies (22).

Data on SARS-CoV-2-specific T cell immunity in patients with primary antibody deficiency (PAD) is still rare. (Cross) reactive T cells against other endemic human coronaviruses were found in CVID patients (23), and a group of five mild COVID-19 cases in PAD patients, with largely preserved specific antibody response, was recently reported to generate a robust T cell response (24). In addition, studies in PAD patients reported detectable specific T cell responses after COVID-19 vaccination (25–28).

Analyzing T cellular and innate immune response in severe COVID-19 in PAD patients without specific humoral immunity may help to identify necessary and redundant aspects of immune response to COVID-19 and is important to understand the different clinical outcomes in patients with antibody deficiency.

In the present study, we report clinical and immunological findings of 5 PAD patients with severe COVID-19 that failed to generate a specific humoral immune response to SARS-CoV-2. All patients presented with respiratory insufficiency due to COVID-19 pneumonia and two patients developed a fatal disease course. SARS-CoV-2 reactive T cells to spike (S) and nucleocapsid (NCAP) peptide pools were analysed comparatively by flow cytometry in PAD patients, convalescents, and naïve healthy individuals. We analyzed innate immune response by assessing CD169 (also termed: sialic acid-binding immunoglobulin-like lectin 1 or SIGLEC1) expression on monocytes. The activation of the type I IFN pathway results in a rapid increase of SIGLEC1 expression on the surface of macrophages and monocytes. Hence, type I IFN levels correlate with expression of SIGLEC1 on monocytes (29). In addition, presence of type I IFN autoantibodies was examined. Clinical and virological outcomes after COVID-19 convalescent plasma (CP) and monoclonal antibody therapy in three PAD patients with RNAemia are presented.

## Methods

### Study Subjects

Five patients with severe COVID-19 and confirmed diagnosis of CVID (n:3; fulfilling the European Society for Immunodeficiencies (ESID) criteria) and Good’s syndrome (n:2), six mild COVID-19 convalescent healthy controls (CHC) and naïve healthy controls (HC) were included (**Table 1**). All cases were diagnosed by RT-PCR in nasopharyngeal swab. All patients, CHC and HC were not vaccinated against COVID-19. Blood samples from naïve HCs were drawn from the laboratory staff. Those naïve controls did not have a history of prior SARS-CoV-2 infection. Our study was approved by the Ethics Committee of Charité Universitätsmedizin Berlin in accordance with the 1964 Declaration of Helsinki and its later amendments (EA2/092/20 from June 4, 2020). All patients and controls gave written informed consent.

**Table 1:** Baseline characteristics of COVID-19 PAD patients.

	Age	Sex	Underlying PAD	Comorbidities	IgG in g/l (before IgRT)	IgA g/l	IgM g/l	CD4^+^/nl	CD8^+^/nl	NK cells/nl	CD19^+^/nl	csmBc (in % of CD19+)
**Patient 1**	56	m	CVID	GLILD, cachexia (BMI: 16), thalassemia minor, low dose steroid treatment (2.5mg/d)	0.0	<0.1	<0.05	0.36	0.72	0.08	0.10	1.1
**Patient 2**	48	f	CVID	Bronchiectasis, DM type 2, thalassemia minor	3.2	<0.1	<0.05	0.60	1.17	0.08	0.01	–
**Patient 3**	49	m	CVID	obesity (BMI 31.2)	0.97	<0.1	<0.05	0.59	0.34	0.06	0.34	0.0
**Patient 4**	43	m	Good’s syndrome	none (thymectomy due to thymoma in 2016)	6.7	1.2	<0.05	0.37	0.31	0.26	0.00	–
**Patient 5**	49	m	Good’s syndrome	New diagnosed and untreated thymoma	0.65	0.15	0.07	0.72	2.17	0.08	0.00	–

BMI, body mass index; csmBc, class-switched memory B cells; CVID, common variable immunodeficiency disorder; DM, diabetes mellitus; f, female; GLILD, granulomatous–lymphocytic interstitial lung disease; IgRT, immunoglobulin replacement therapy; m, male.

### SARS-CoV-2 Antibody Serology

SARS-CoV-2 IgG against the S1 and N-terminal domain of SARS-CoV-2 spike was analyzed in serum by ELISA according to the manufacturer’s instructions (Euroimmun Medizinische Labordiagnostika AG, Lübeck, Germany) and also by using fully automated Euroimmun Analyzer I (Euroimmun Medizinische Labordiagnostika AG, Lübeck, Germany). A positive IgG and IgA antibody response was determined by optical density (OD) ratios above 1.1. Titer of SARS-CoV-2 neutralizing IgG was assessed by the use of plaque reduction neutralization test (PRNT) as it has been previously described (30).

### SARS-CoV-2 RT-PCR in Plasma

RT-PCR testing for SARS-CoV-2 RNA in plasma or serum samples was done as previously described (31) and by using a preformulated oligonucleotide mixture (Tib-Molbiol, Berlin, Germany).

### CD169/SIGLEC1 Expression on Monocytes

In all severe COVID-19 PAD patients, SIGLEC1 expression on monocytes was analyzed in EDTA whole blood based on a method described previously (32). SIGLEC1 levels on monocytes were investigated using an admitted flow cytometry protocol at the clinical diagnostics laboratory (Labor Berlin GmbH). Samples were stained with mouse anti-human antibodies against SIGLEC1 (clone 7–239), CD14 and CD45 (antibodies purchased from Beckman Coulter).

### Detection of Anti-Type I IFN Autoantibodies

Presence of anti-type I IFN autoantibodies was analyzed using an electrochemiluminescence immunoassay-platform (MSD, Rockville, U.S.) as described previously (33). Light signal count (LSC) levels <1,980 for anti-Interferon-α antibodies and LSC levels <1,961 for anti-interferon-ω antibodies are considered negative.

### Generation of Convalescent Plasma

Plasma was collected from convalescent COVID-19 patients with confirmed neutralizing antibody titer in PRNT50 of at least 1:320 (21) and fulfilling all national standards for blood donation. Single-donor apheresis was conducted with Trima Accel System (Automated Blood Collection) Terumo BCT, Inc. Depending from body weight, two to three bags of 220 ml each were collected and frozen rapidly at <−30°C in less than 8 h after collection. Dosing of CP was conducted to achieve an expected PRNT50 of ≥1:40 in the recipient.

### Cell Isolation and Culture

Time point of sampling for T cell analysis is indicated in **Table 2**. Patients were off Dexamethasone treatment at least 10 days prior to sampling. Median time post symptom onset in healthy convalescent controls was 87 days (60–157 days). Peripheral blood mononuclear cells (PBMCs) were isolated from heparinized whole blood using density gradient centrifugation and transferred to liquid nitrogen. PBMCs were thawed and rested for 24 h in IMDM medium supplemented with 10% fetal calf serum and 1% penicillin/streptomycin. Cells were seeded with a concentration of 2 × 10^6^/ml. The background control was incubated with DMSO only and the positive control was stimulated with 3 µg/ml superantigen staphylococcal enterotoxin B (SEB). Stimulation for SARS-CoV-2 responsive T cells was performed using 1 µg/ml of peptide pools for S (two vials with N-term and C-term, PM-WCPV-S-1) and NCAP (PM-WCPV-NCAP-1) proteins (JPT Peptide Technologies GmbH, Berlin). Samples were incubated for 18 h under standard conditions (37°C, 5% CO_2_). After 2 h of incubation with peptide pools, brefeldin A (BFA), a secretion inhibitor, was added to the cell culture.

**Table 2:** Clinical and laboratory parameters specific for COVID-19 of PAD patients.

	Underlying PAD	RT-PCR in nasal swab	RT-PCR in peripheral blood	Spike-IgG and -IgA	SIGLEC1 [molecules/monocyte] (norm. <2,400)	Clinical severity according to WHO R&D blueprint scale	COVID-19 treatment	Clinical outcome	Duration of RT-PCR positivity in nasal swab [d]	Time point of blood sampling for T cell response after symptom onset [d]
Patient 1	CVID	pos.	pos. (max. viral load: 3.2 × 10^4^ copies/ml)	neg.	9,771	6→8	dexamethasone, IVIG, CP	deceased	fatal COVID-19 (40)	39
Patient 2	CVID	pos.	neg.	neg.	15,485	4	dexamethasone, IVIG,	recovered	62	40
Patient 3	CVID	pos.	neg.	neg.	11,741	4	dexamethasone	recovered	61	24
Patient 4	Good’s syndrome	pos.	pos. (max. viral load: 7 × 10^4^ copies/ml)	neg.	1,423	5	dexamethasone, IVIG, CP	recovered	127	128
Patient 5	Good’s syndrome	pos.	pos. (max. viral load: 8.8 × 10^4^ copies/ml)	neg.	11,758	7→8	dexamethasone, IVIG, mAb	deceased	47	not done

CP, convalescent plasma; CVID, common variable immunodeficiency disorder; IVIG, intravenous immunoglobulin; mAb, monoclonal antibodies; neg., negative; pos, positive; SIGLEC1, sialic acid-binding immunoglobulin-like lectin 1.

### Detection of SARS-CoV-2 Antigen Specific T Cells

PBMCs were stained on their surface with LIVE/DEAD Fixable Blue Dead Cell Stain Kit (Thermo Fisher Scientific) following fixation and permeabilization (FoxP3 transcription factor staining buffer set, eBioscience). Staining for intracellular markers was conducted using human anti-CD3 BV650 (OKT3), -CD4 PerCp-Cy5.5 (SK3), -CD8 BV510 (RPA-T8), -CD137 PE (4B4-1), CD154 BV421 (24–31), IL-2 AP (MQ1-17H12), -IFNγ BV605 (4S.B3), -TNFα AF700 (MAb11) (Biolegend). Samples were analyzed using CytoflexLX Flow Cytometer (Beckman Coulter) and FlowJo software (version 10.6.2, BD). A positive T cell response was defined as frequency of CD154^+^CD137^+^CD4^+^ T cells being ≥0.005% within total CD4^+^ T cells. Moreover, samples signals had to exceed the background signal by a minimum of 20%. This threshold value refers to the range in which 95% of all negative samples are. In order to exclude unspecific activation the background signal (DMSO sample) was subtracted from the peptide stimulated samples. Frequencies of effector cytokine producing antigen specific T cells were analyzed for IFNγ, TNFα, IL-2 and seven different subsets were examined by Boolean combination gating strategy to identify single (sp), double (dp) or triple (tp) cytokine producing activated T cell populations.

### Statistical Analyses

Data analyses was performed using GraphPad Prism 6 software. Nonparametric statistical methods were used. Continuous variables were expressed as median and interquartile range (IQR). Univariate comparisons of T cell responses in two independent groups were performed using Mann–Whitney-U test. A p-value of <0.05 was considered statistically significant. Because of multiple testing p-values are considered descriptive.

## Results

### Study Subjects and Humoral Immune Response

PAD patients fulfilled ESID criteria for CVID (n:3) and Good’s syndrome (n:2) (see **Table 1** for detailed patient baseline characteristics). All five PAD patients had SARS-CoV-2 infections confirmed by RT-PCR in nasopharyngeal swab. Severity was graded according to the Centre of Disease Control (34). Extensive bilateral COVID-19 pneumonia was detected by chest CT in four cases (patient #1, #3, #4, #5), with onset of respiratory insufficiency occurring between days 8 and 20 PSO. Patient #2 had milder respiratory insufficiency (SpO2: 91% at room air) together with infiltrates in chest X-ray. Please refer to **Table 2** and **Supplementary Text 1** for detailed clinical case descriptions.

All PAD patients (patient #1–5) failed to mount a specific antibody response to SARS-CoV-2 (negative for IgG and IgA antibodies) (**Table 2**). Serological response in RT-PCR-confirmed COVID-19 convalescent cohort showed positive SARS-CoV-2-IgG in three, borderline positive tests in two and a negative test result in one patient. SARS-CoV-2-IgA was positive in five individuals, while one convalescent individual was tested negative. As expected, SARS-CoV-2-IgG and -IgA was not detected in healthy controls (**Supplementary Figure 1**).

### PAD Patients With COVID-19 Show High Frequencies of SARS-CoV-2 Specific T-Cells

For specific T cell responses we analyzed 4 PAD patients, 6 CHC and 6 naïve HC. For detailed gating strategy please refer to **Supplementary Figure 2**.

**Figure 1:**
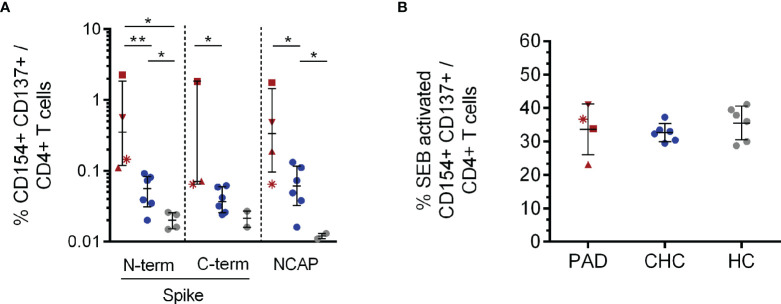
CD154^+^CD137^+^CD4^+^ T cell response to SARS-CoV-2 Spike and NCAP peptide pools and SEB positive control. PBMCs of PAD (n = 4, red), CHC (n = 6, blue) and naïve HC (n = 6, grey) were stimulated with 1 µg/ml SARS-CoV-2 peptides or 3 µg/ml SEB. Frequencies of activated CD154^+^CD137^+^CD4^+^ **(A)** T cells after stimulation with the different SARS-CoV-2 peptides and SEB **(B)**. Only T cell responses above the threshold of 20% above background activation are shown. Median and interquartile range (IQR) are indicated. Statistical analysis was performed by non-parametric one-tailed Mann–Whitney-U test for comparison of control and patient groups. A p-value ≤0.05 was considered as statistically significant. p ≤0. 05 = *; p ≤0.001 = **. PAD patients are depicted by the following symbols: patient #1 *; patient #2 ▲; patient #3 ■; patient #4 ▼.

All analyzed PAD patients had SARS-CoV-2 S reactive CD154^+^CD137^+^CD4^+^ T cells either to the N- (n:4) or C-terminal (n:3) part. 6/6 CHC had reactive CD4^+^ T cells to both SARS-CoV-2 S peptide pools, whereas naïve HCs had fewer N- (n = 4) and C-terminal (n = 2) responses. PAD patients showed significantly higher frequencies of CD4^+^ antigen specific T cells to the N-terminus compared to CHC (p = 0.005) and naïve HC (p = 0.014) (**Figure 1A**). CHC also showed significantly higher Spike N-terminal reactive CD4^+^ T cells compared to naïve HC (p = 0.02). Regarding C-terminal S reactive CD4^+^ T cells, PAD patients had higher frequencies compared to CHC (p = 0.02). Moreover, PBMC stimulation with SARS-CoV-2 NCAP peptide pool elucidated higher frequencies of antigen specific CD4^+^ T cells in 4/4 PAD patients compared to CHC (p = 0.03) and also in CHC compared to naïve HC (p = 0.04) (**Figure 1A**). Stimulation with the positive control SEB resulted in similar frequencies of CD4^+^ activated T cells among the investigated groups (**Figure 1B**).

Patient #1 already expressed SARS-CoV-2-S-reactive T cells seven months prior to his severe COVID-19 disease as reported in our previous study on SARS-CoV-2 cross-reactive T cells in CVID patients (23). Before SARS-CoV-2 infection patient #1 only expressed low levels of activated T cells (CD154^+^CD137^+^CD4^+^) reactive to N-terminus of SARS-CoV-2 S protein and also against S of human endemic corona viruses. During severe COVID-19 disease, activated CD4^+^ T cells reactive to SARS-CoV-2 C-terminal S and NCAP were also detectable and frequencies of activated, reactive T cells were generally much higher (1.8–2.2% CD154^+^CD137^+^CD4^+^ during COVID-19) (**Supplementary Table 1**).

CD8^+^ T cell responses to peptide pools of SARS-CoV-2 structural proteins were observed in all 4 PAD patients. However, frequencies showed no differences compared to CHC and HC (**Supplementary Figure 3A**). SEB stimulation serving as positive control resulted in comparable frequencies of activated CD137^+^CD8^+^ T cells among the investigated groups (**Supplementary Figure 3B**).

### SARS-CoV-2 Antigen Specific CD4^+^ T Cells of PAD Patients and Convalescent Healthy Controls Express a Distinct Cytokine Profile

**Figure 2:**
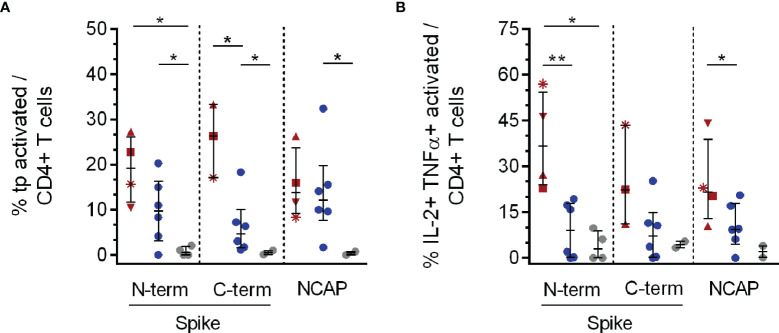
Triple and double cytokine producing activated CD4^+^ T cell subsets in response to SARS-CoV-2 specific peptide stimulation. PBMCs of PAD (n = 4, red), CHC (n = 6, blue) and naïve HC (n = 6, grey) were stimulated with 1 µg/ml SARS-CoV-2 peptides or 3 µg/ml SEB. IFNγ, TNFα and IL-2 tp activated CD4^+^ T cells were analyzed by Boolean combination gating strategy. Cytokine expression profile in tp activated CD4^+^CD154^+^CD137^+^ **(A)** T cells, and also TNFα + IL-2 dp **(B)** activated CD4^+^ T cells in response to SARS-CoV-2 peptide pools are shown. Median and interquartile range (IQR) are indicated. Statistical analysis was performed by non-parametric one-tailed Mann–Whitney-U test for comparison of control and patient groups. A p-value ≤0.05 was considered as statistically significant. p ≤0. 05 = *; p ≤0.001 = **. PAD patients are depicted by the following symbols: patient #1 *; patient #2 ▲; patient #3 ■; patient #4 ▼.

In all analyzed PAD patients and CHC we observed triple-positive (tp) antigen specific CD4^+^ T cells following stimulation with SARS-CoV-2 Spike (S) N- and C-terminal peptide pools (see **Figure 2A** and **Supplementary Figure 2** for gating strategy and representative flow cytometry plots). PAD patients showed a higher expression of triple cytokine producing activated CD4^+^ T cells compared to CHC (C-terminal, p = 0.02) and naïve HC (N-terminal, p = 0.014) in response to SARS-CoV-2 S. Frequencies of S- and NCAP-reactive triple cytokine producing activated CD4^+^ T cells were significantly higher in CHC compared to HC (N-terminal, p = 0.03; C-terminal, p = 0.04) (**Figure 2A**). CHC further expressed higher NCAP reactive triple cytokine producing activated CD4^+^ T cells compared to HC (p = 0.04). Overall highest responses to specific SARS-CoV-2 stimulation were observed for TNFα + IL-2 double cytokine producing activated CD4^+^ T cells with PAD patients showing significantly higher S N-terminal specific TNFα + IL-2 dp CD4^+^ T cells compared to CHC (p = 0.014) and HC (p = 0.005). This was as well observed for PAD patients compared to CHC (p = 0.03) after NCAP specific stimulation (**Figure 2B**). T cell responses for the remaining single and double cytokine producing CD4^+^ T cell subsets did not show a distinct pattern and are shown in **Supplementary Figure 4**.

CD8^+^ T cells showed no differences in frequencies of cytokine reactive T cells (**Supplementary Figures 5A–G**).

### Detection of Viral Load in Blood, Prolonged Viral Shedding and Treatment With Specific SARS-CoV-2 Antibodies in PAD Patients

In three severe cases of COVID-19 (patient #1, #4 and #5), SARS-CoV-2-RNA was detected in peripheral blood with 3.2 × 10^4^, 7 × 10^4^ and 8.8 × 10^4^ copies/ml respectively (**Table 2**). Transfusion of 440 ml AB0-compatible COVID-19 CP with confirmed levels of neutralizing antibodies (PRNT50 ≥1:320) was conducted in patient #1 and #4 and was well tolerated. Patient #5 received 8 g of Casirivimab/Imdevimab. Rapid decline of SARS-CoV-2 viral load in peripheral blood was observed after CP treatment within 3 days post treatment. Decrease of viral load in peripheral blood was seen 6 days after treatment with monoclonal antibodies Casirivimab/Imdevimab and RT-PCR in plasma turned negative after 14 days. Serological follow-up showed detectable SARS-CoV-2-specific IgG (#1, #4, #5) and IgA (#1 and #4) respectively (see **Table 3**).

**Table 3:** SARS-CoV-2 antibody serology in serum and viral load in peripheral blood of PAD patients treated with convalescent plasma and monoclonal antibodies.

	SARS-CoV-2-Spike-IgG ratio	SARS-CoV-2-Spike-IgA ratio	PRNT50	SARS-CoV-2 RT-PCR in peripheral blood
Patient 1 (before 1st CP treatment)	0.18 (neg.)	0.06 (neg.)	<1:20 (neg.)	pos. (3.2 × 10^4^ copies/ml)
Patient 1 (d1 after 1st CP treatment)	2.16 (pos.)	0.37 (neg.)	1:20	pos. (6.1 × 10^3^ copies/ml)
Patient 1 (d3 after 1st CP treatment)	1.16 (pos.)	1.3 (pos.)	not done	neg.
Patient 1 (before 2nd CP treatment)	0.49 (neg.)	0.08 (neg.)	not done	neg.
Patient 1 (d2 after 2nd CP treatment)	1.88 (pos.)	1.16 (pos.)	1:40	not done
Patient 4 (before CP treatment)	0.07 (neg.)	0.39 (neg.)	<1:20 (neg.)	pos. (7 × 10^4^ copies/ml)
Patient 4 (d1 after CP treatment)	4.22 (pos.)	1.08 (borderline pos.)	1:80	not done
Patient 4 (d3 after CP treatment)	2.43 (pos.)	0.42 (neg.)	not done	<500 copies/ml (negative)
Patient 5 (before mAb treatment)	0.04 (neg.)	0.06 (neg.)	not done	pos. (8.8 × 10^4^ copies/ml)
Patient 5 (d6 after mAb treatment)	10.94 (pos.)	0.18	not done	pos. (5 × 10^3^ copies/ml)
Patient 5 (d14 after mAb treatment)	>11 (pos.)	0.21	not done	neg.

CP, convalescent plasma; mAb, monoclonal antibody; neg., negative; pos., positive.

Prolonged viral shedding (>40 days) was confirmed by RT-PCR in nasopharygeal swabs in all patients with patient #4 remaining positive for 127 days PSO (**Table 2**).

### Innate Immune Response by SIGLEC1 Expression on Monocytes and Anti-Type I IFN Autoantibodies

SIGLEC1 on monocytes is a downstream molecule in IFN signaling serving as a surrogate marker of type I IFN signature (35). Apart from patient #4, all patients had elevated levels of SIGLEC on monocytes, indicating a normal type I IFN response. Patient #4 expressed low levels of SIGLEC1 (1,423 molecules/monocyte) despite RNAemia (**Table 2**). However, no autoantibodies against IFN-α or IFN-ω were detected in any of our patients (**Supplementary Table 2**). Reanalysis of previously conducted whole exome sequencing of patient #1 revealed a heterozygous mutation in *IFNAR1* (V307I), which was reported to be of no functional relevance [(12) and personal communication with JL Casanova and Q Zhang]. WES data of patient #3 did not reveal any suspect or disease causing mutations. In the remaining patients WES was not conducted.

## Discussion

In the present study of severe COVID-19 cases in PAD patients with undetectable SARS-CoV-2-specific antibodies, a robust T cell response was observed. Similar to convalescent immunocompetent patients, where polyfunctional SARS-CoV-2-specific T cells have been described (36), triple cytokine-producing, activated T cells were observed in our PAD patients, indicating the generation of a memory-like phenotype (36, 37). T cell response to COVID-19 was even stronger in PAD patients than in convalescent healthy controls (CHC), which is probably due to the generally milder disease course and slightly later median time point of sampling in the control group.

Innate immune response by type I IFN was shown to be of pivotal importance, and patients with defects in type I IFN signaling or autoantibodies against interferon-α or -ω are at increased risk for severe COVID-19 (12, 13). In IEI patients, type I IFN autoantibodies were reported for APS-1 patients (14). Although described previously (38, 39), impact of type I IFN autoantibodies on the high case fatality rate in Good’s syndrome was never examined.

By assessing SIGLEC1 on monocytes, a robust *ex vivo* marker of type I IFN response, we observed an expected rise of expression levels in 4/5 patients. Low levels in patient #4 (Good’s syndrome) and decline of SIGLEC1 expression levels in patient #1, despite RNAemia, prompted evaluation of anti-cytokine antibodies. However, we could not find anti-IFN-α or -IFN-ω autoantibodies in any of our severe COVID-19 cases. Decreasing SIGLEC-1 expression during disease course might be related to concurrent medication with dexamethasone.

SARS-CoV-2 viral load, and in particular viral load in blood, was shown to be associated with increased risk of mortality (40, 41). In the here reported severe COVID-19 patients without specific antibodies but detectable RNAemia, viral clearance in blood, and in part also clinical improvement, was clearly associated with administration of specific antibodies in convalescent plasma (CP) and monoclonal antibodies. COVID-19 CP has been used previously in IEI patients and its efficacy is usually evaluated clinically and according to viral load in nasopharyngeal or respiratory tract specimens (42), but evaluation of virus neutralizing capacity in blood in IEI patients is missing (17, 43, 44).

It is unknown, whether a lack of specific humoral immunity to SARS-CoV-2 per se leads to an increased risk of RNAemia. Based on numerous mild COVID-19 cases in XLA patients (2, 15–20), at least generally persisting and high viral loads seem unlikely.

Data obtained during acute SARS-CoV-2 infection from larger cohorts of mild and severe COVID-19 cases in antibody deficient patients are needed to approach this question. Our data suggest a more differentiated approach to CP and SARS-CoV-2 monoclonal antibody treatment, which should include testing of viral load in blood and assessing antibody titer in the plasma recipient in order to detect accelerated reduction of transfused antibody levels related to virus neutralization and antibody consumption.

Despite a robust T cell response, we observed prolonged viral shedding > 40 days after initial positive RT-PCR in all our patients. Patient #4, suffering from Good’s syndrome, even remained positive for 127 days. Prolonged viral shedding of SARS-CoV-2 in IEI patients was reported previously (2). Although longer periods of persisting viral shedding are also reported in the general (45) and elderly seropositive population (46), the extended duration of viral shedding in primary and secondary antibody deficiency suggests a role for antibodies in viral clearance. Of note, prolonged shedding was shown to be associated with marked within-host genomic evolution of SARS-CoV-2 with continuous turnover of dominant viral variants (47), warranting prolonged surveillance and testing of seronegative PID patients with COVID-19.

Overall, our observations of a robust T cellular immunity and detectable type I IFN innate response in severe COVID-19 patients without specific SARS-CoV-2 antibodies support the relevance of humoral immunity.

Our study included one CVID patient where SARS-CoV-2-reactive T cells had been described prior to his COVID-19 disease (23). These preexisting cross-reactive T cells to SARS-CoV-2, were described in 35–90% of unexposed healthy individuals (36, 37, 48–52), however their role during infection is a matter on ongoing debate. At least in our patient, pre-existing reactive T cells in the absence of specific SARS-CoV-2 antibodies did not prevent severe COVID-19. However, the level of preexisting T cell immunity was rather low and increased substantially during infection. Therefore this observation does not allow to draw conclusions on possible protective levels of T cell immunity after vaccination.

As shown previously for XLA and CVID patients after vaccination against seasonal influenza or hepatitis B (53–55), induction of specific T cell immunity is also detectable after COVID-19 vaccination in IEI patients (25–28).

The increased levels of NCAP-reactive T cells suggest that patients unable of generating specific antibodies, might possibly benefit from vaccines immunizing against spike and nucelocapsid structures.

Having in mind the recent data on prophylactic monoclonal antibody treatment (56, 57), our observations support the use of prophylactic antibody therapy in patients with known failure to mount a specific antibody response against SARS-CoV-2.

Although current data indicate, that commercially available immunoglobulin products already contain neutralizing SARS-CoV-2 antibodies to a certain extent (58), neutralizing SARS-CoV-2 antibodies might not be detectable in commercially available products before Spring/Summer 2022 due to the long production process and safety regulations. Clinical effectiveness of SARS-CoV-2-specific antibodies in immunoglobulin products against present and emerging SARS-CoV-2 variants remain to be investigated.

## Data Availability Statement

The raw data supporting the conclusions of this article will be made available by the authors, without undue reservation.

## Ethics Statement

The studies involving human participants were reviewed and approved by the Ethics Committee of Charité Universitätsmedizin Berlin in accordance with the 1964 Declaration of Helsinki and its later amendments (EA2/092/20 from June 4, 2020). The patients/participants provided their written informed consent to participate in this study.

## Author Contributions

LH and CS made substantial contributions to conception and design of the study and lead the project. LH, MK, AW, SG, JK, DZ, KW, and AP made patient samples available. NA, UK and APr made convalescent plasma available. SS planned and performed all experiments on T cell assays, analyzed results, composed all figures and interpreted data. SS and LH wrote the manuscript. LG and SB provided support during experimental implementation. SS, CS, and LH interpreted data. VC, TS, and CD and performed analysis of SARS-CoV-2 serology. CM, OS, and TM analyzed type I IFN autoantibodies. All authors listed have made a substantial, direct, and intellectual contribution to the work and approved it for publication.

## Funding

We acknowledge support from the German Research Foundation (DFG) and the Open Access Publication Fund of Charité - Universitätsmedizin Berlin.

## Conflict of Interest

VC is named together with Euroimmun on a patent application filed recently regarding detection of antibodies against SARS-CoV-2. Authors CM, TM and OS are employed by Labor Berlin GmbH.

The remaining authors declare that the research was conducted in the absence of any commercial or financial relationships that could be construed as a potential conflict of interest.

## Publisher’s Note

All claims expressed in this article are solely those of the authors and do not necessarily represent those of their affiliated organizations, or those of the publisher, the editors and the reviewers. Any product that may be evaluated in this article, or claim that may be made by its manufacturer, is not guaranteed or endorsed by the publisher.

## Supplementary Material

Supplementary Figure 1 SARS-CoV-2 antibody serology in COVID-19 PAD patients, convalescent (CHC) and naïve (HC) healthy controls. Serum IgG **(A)** and IgA **(B)** against the N-terminal domain of the spike protein, including the immunologically relevant receptor binding domain (RBD) of SARS-CoV-2, were analyzed by EUROIMMUN Anti-SARS-CoV-2 ELISAs. Results are evaluated by calculating a ratio of the OD of the control or patient sample over the OD of a calibrator. A ratio <0.8 is negative, ratio ≥ 0.8 to <1.1 is borderline, ratio ≥1.1 is positive (dotted lines). Median and interquartile range (IQR) are indicated. Statistical analysis was performed by non-parametric two-tailed Mann–Whitney-U test for comparison of control and patient groups. A p-value ≤ 0.05 was considered as statistically significant. p ≤ 0.05 = *; p ≤ 0.005 = **. PAD patients are depicted by the following symbols: patient #1 *****; patient #2 ▲; patient #3 ■; patient #4 ▼, patient #5 ♦.

Supplementary Figure 2 Representative flow cytometry plots for analysis of activated CD4+ and CD8+ T cells and their expression of effector cytokines. Example of a gating strategy in presence of a specific stimulation with SARS-CoV-2 Spike N-terminal peptide pool. PBMCs were gated on lymphocytes. Douplets as well as dead cells were excluded. Living CD3+ T cells were distinguished in CD4+ helper and CD8+ effector T cells. Within those T cell populations activated CD4+CD154+CD137+ and CD8+CD137+ T cells were gated and the expression of IFNγ, TNFα and IL-2 analyzed. Single, double or triple cytokine producing activated T cell subsets were analyzed using Boolean combination gates.

Supplementary Figure 3 CD137+CD8+ T cell response to SARS-CoV-2 Spike and NCAP peptide pools and SEB positive control. PBMCs of PAD (n=4, red), CHC (n=6, blue) and naïve HC (n=6, grey) were stimulated with 1 µg/ml SARS-CoV-2 peptides or 3 µg/ml SEB. Frequencies of activated CD137+ CD8+ T cells **(A)** after stimulation with the different SARS-CoV-2 peptides. Frequencies of activated CD137+ CD8+ T cells **(B)** after stimulation with SEB. Only T cell responses above the threshold of 20% above background activation are shown. Median and interquartile range (IQR) are indicated. Statistical analysis was performed by non-parametric one-tailed Mann–Whitney-U test for comparison of control and patient groups. A p-value ≤ 0.05 was considered as statistically significant. PAD patients are depicted by the following symbols: patient #1 *****; patient #2 ▲; patient #3 ■; patient #4 ▼.

Supplementary Figure 4 Single and double cytokine producing activated CD4+ T cells in response to SARS-CoV-2 specific peptide stimulation. IFNγ, TNFα, or IL2 single (sp) or double (dp) producing activated CD4+ T cells were analyzed by Boolean combination gating strategy. IFNγ **(A)**, TNFα **(B)** and IL2 **(C)** sp and IFNγ+IL-2+ **(D)** and IFNγ+ TNFα **(E)** dp activated CD4+ T cells in response to SARS-CoV-2 peptide pools are shown. Median and interquartile range (IQR) are indicated. Statistical analysis was performed by non-parametric one-tailed Mann–Whitney-U test for comparison of control and patient groups. A p-value ≤ 0.05 was considered as statistically significant. p ≤ 0.05 = *. PAD patients are depicted by the following symbols: patient #1 *****; patient #2 ▲; patient #3 ■; patient #4 ▼.

Supplementary Figure 5 Single, double and triple cytokine producing activated CD8+ T cells in response to SARS-CoV-2 specific peptide stimulation. IFNγ, TNFα, or IL2 single (sp) or double (dp) or triple (tp) producing activated CD8+ T cells were analyzed by Boolean combination gating strategy. IFNγ **(A)**, TNFα **(B)** and IL2 **(C)** sp and IFNγ+IL-2 **(D)**, IFNγ+TNFα **(E)** IL-2+TNFα **(F)** dp and IFNγ+TNFα+IL2 tp activated CD8+ T cells in response to SARS-CoV-2 peptide pools are shown. Median and interquartile range (IQR) are indicated. Statistical analysis was performed by non-parametric one-tailed Mann–Whitney-U test for comparison of control and patient groups. A p-value ≤ 0.05 was considered as statistically significant. PAD patients are depicted by the following symbols: patient #1 *; patient #2 ▲; patient #3 ■; patient #4 ▼.

Supplementary Table 1 Activated CD4+ T cell frequencies and cytokine response of patient #1 before and during SARS-CoV-2 infection. Please refer to Excel sheet **Table 1**.

Supplementary Table 2 Light signal count (LSC) for anti-IFN α and -ω autoantibodies determined by electrochemiluminescence immunoassay-platform.

Supplementary Text 1 Detailed clinical case descriptions of PAD Patients.
