# Supplementary figures and images for: SARS-CoV-2 T Cell Response in Severe and Fatal COVID-19 in Primary Antibody Deficiency Patients Without Specific Humoral Immunity
Source: Front Immunol. 2022 Mar 10;13:840126. doi: 10.3389/fimmu.2022.840126 (PMC8960624; doi:10.3389/fimmu.2022.840126)

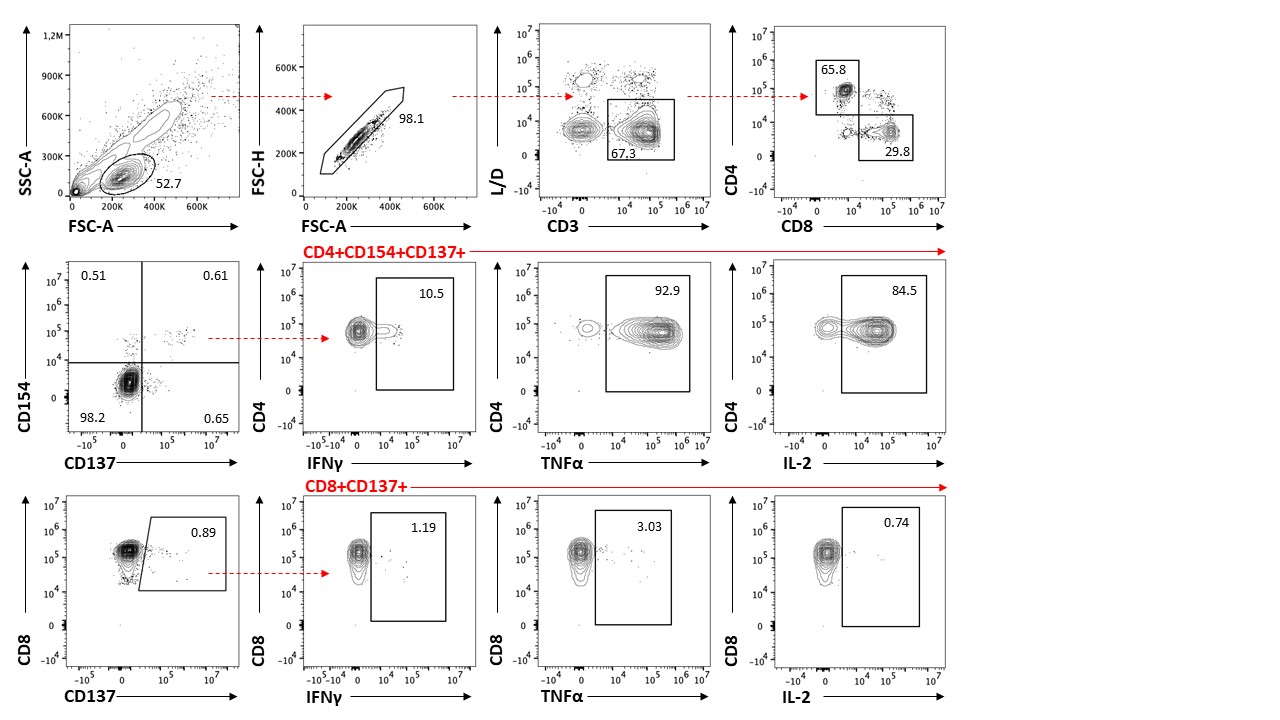

Supplement: Supplementary Figure 2 — Representative flow cytometry plots for analysis of activated CD4+ and CD8+ T cells and their expression of effector cytokines. Example of a gating strategy in presence of a specific stimulation with SARS-CoV-2 Spike N-terminal peptide pool. PBMCs were gated on lymphocytes. Douplets as well as dead cells were excluded. Living CD3+ T cells were distinguished in CD4+ helper and CD8+ effector T cells. Within those T cell populations activated CD4+CD154+CD137+ and CD8+CD137+ T cells were gated and the expression of IFNγ, TNFα and IL-2 analyzed. Single, double or triple cytokine producing activated T cell subsets were analyzed using Boolean combination gates. [file Image_2.jpeg]
