# Supplementary material for: SARS-CoV-2 T Cell Response in Severe and Fatal COVID-19 in Primary Antibody Deficiency Patients Without Specific Humoral Immunity
Source: Front Immunol. 2022 Mar 10;13:840126. doi: 10.3389/fimmu.2022.840126 (PMC8960624; doi:10.3389/fimmu.2022.840126)
Supplement: Supplementary Table 2 — Light signal count (LSC) for anti-IFN α and -ω autoantibodies determined by electrochemiluminescence immunoassay-platform. [file Table_2.docx]

| **Patient** | **alpha Biotynilation** | **light signal count (LSC) IFN-alpha antibody** | **omega Biotynilation** | **light signal count (LSC) IFN-omega antibody** |
| --- | --- | --- | --- | --- |
| Patient #1 | 1:1000 | 129 (negative) | 1:500 | 141 (negative) |
| Patient #2 | 1:1000 | 162 (negative) | 1:500 | 96 (negative) |
| Patient #3 | 1:1000 | 72 (negative) | 1:500 | 78 (negative) |
| Patient #4 | 1:1000 | 333 (negative) | 1:500 | 108 (negative) |
| Patient #5 | 1:1000 | < 1980 (negative) | 1:500 | < 1961 (negative) |

**Supplementary table 2:** Light signal count (LSC) for anti-interferon-alpha and -omega autoantibodies determined by electrochemiluminescence immunoassay-platform.
