# Supplementary material for: SARS-CoV-2 T Cell Response in Severe and Fatal COVID-19 in Primary Antibody Deficiency Patients Without Specific Humoral Immunity
Source: Front Immunol. 2022 Mar 10;13:840126. doi: 10.3389/fimmu.2022.840126 (PMC8960624; doi:10.3389/fimmu.2022.840126)
Supplement: Supplementary Text 1 — Detailed clinical case descriptions of PAD Patients. [file DataSheet_1.docx]

**Online Supplementary Text 1: Detailed clinical case descriptions of PAD patients**

**Patient #1**

Patient #1 is a 56-year old male patient of Libanese provenance with CVID (EUROclass smB-21low) diagnosed 6 years earlier. Under immunoglobulin replacement therapy (IgRT) patient 1 was clinically stable with no frequent respiratory infections despite mild GLILD. Patient #1 suffered from recurrent *Campylobacter jejuni* infections with weight loss (BMI: 16) and thalassemia minor.

Patient #1 was tested positive for SARS-CoV-2 in nasal swab by RT-PCR in October 2020. He presented initially with fever and gastrointestinal symptoms and was admitted to hospital at day 3 post symptom onset (PSO). Chest CT showed bilateral pneumonia. Day 8 PSO he developed respiratory insufficiency requiring supplemental oxygen. Treatment with high dose IVIG and Dexamethasone was initiated. Despite broad antimicrobial treatment due to suspected bacterial superinfection, respiratory condition deteriorated and patient was admitted to ICU for high flow oxygen treatment at day 22 PSO. SARS-CoV-RT PCR detected viral load in peripheral blood (3,2x10^4^ copies/ml) and remained positive in sputum without presence of specific SARS-CoV-2 antibodies at day 22 PSO. Therefore 440ml of convalescent plasma (including neutralizing antibodies tested in PRNT50 with 1:320) was administered at day 23 PSO and resulted in viral clearance in peripheral blood at day 26 PSO. With continued requirement for high flow oxygen treatment, patient #1 showed only a mild clinical improvement. In the context of low neutralizing antibody titer, another 440ml of COVID-19 convalescent plasma was infused. Serological data showed an expected rise of SARS-CoV-2-IgG and –IgA antibodies and patients (see table 3) respiratory condition improved from 40 l/min oxygen via highflow to 4 l/min via nasal cannula within 3 days. Viral detection in nasopharyngeal swab persisted. At day 37 PSO patient #1 developed a massive intracerebral hemorrhage and died at day 40 PSO despite immediate neurosurgical intervention.

Post mortem analysis excluded presence of type I interferon autoantibodies. Reanalysis of previously conducted whole exome sequencing revealed a heterozygous mutation in *IFNAR1* (V307I), which was reported to be of no functional relevance (Zhang et al.).

**Patient #2**

Patient #2 is 48-year old female CVID (Euroclass B-) patient of Turkish origin. Patient #2 presented with fever, general fatigue, nausea and vomiting and was tested positive for SARS-CoV-2 by RT-PCR in nasal swab in 11/2020 and 2 days PSO. Patient #2 had mild respiratory symptoms (SpO2:92% at room air) and discrete streaky pattern in chest X-ray. Due to respiratory symptoms supplemental oxygen and treatment with dexamethasone was started at day 8 PSO. Clinical condition improved rapidly, however patient 2 remained positive for SARS-CoV-2 by RT-PCR until day 62 PSO. SARS-CoV-2-Spike-IgG and –IgA were not detectable at any time during COVID-19. Type I interferon autoantibodies were not detected. Presence of SARS-CoV-2 by RT-PCR in peripheral blood was not assessed.

**Patient #3**

Patient 3 is a 49-year old male Caucasian patient with CVID (EUROclass smB-21norm). Patient 3 is under subcutaneous immunoglobulin replacement therapy without relevant infectious or non-infectious complications. Patient 3 presented with fever and diarrhea in January 2021 and was tested positive for SARS-CoV-2 by RT-PCR the same day. On day 14 PSO patient 3 was admitted to hospital with continuing fever up to 40°C and increasing coughing and dyspnea. Bilateral COVID-19 pneumonia was detected by chest CT and supplemental oxygen therapy was initiated at a low flow rate of 3-4 Liters/min. Patient 3 required supplemental oxygen for the following 12 days and was discharged from hospital at day 27 PSO. SARS-CoV-2 RT-PCR resulted negative in peripheral blood but remained positive in nasal swab for 61 days. SARS-CoV-2-Spike-IgG and –IgA were not detectable at any time during COVID-19. Type I interferon autoantibodies were not detected.

**Patient #4**

Patient 4 is a 43-year old Caucasian patient with Good’s syndrome diagnosed 5 years earlier. Patient 4 has a mild IgG reduction, no detectable IgM and a complete loss of CD19+ B cells. Patient 4 presented with symptoms of a common cold and was tested positive for SARS-CoV-2 by RT-PCR in November 2020. At day 20 PSO patient 4 was admitted to hospital due to respiratory insufficiency presenting bilateral pulmonary infiltrates in chest CT. High dose IVIG and dexamethasone had no clinical benefit and patient 4 was transferred to ICU for non-invasive ventilation (NIV).

Patient 4 was tested positive for SARS-CoV-2 in peripheral blood by RT-PCR on day 30 and day 45 PSO. Due to viremia with continuing respiratory insufficiency with requirement for NIV and persistent fever patient 4 received 440ml of COVID-19 convalescent plasma (including neutralizing antibodies tested in PRNT50 with 1:320) on day 46 PSO. Patient 4 improved significantly within 12h after convalescent plasma administration, presenting improved oxygenation and normal temperature. Viral load in peripheral blood declined from 7x10 ^4^ to negative on day 50 PSO.

SARS-CoV-2-Spike-IgG and –IgA were not detectable at any time during COVID-19 but showed an expected increase and detectable neutralizing antibodies after infusion of convalescent plasma. Type I interferon autoantibodies were not detected. Patient 4 remained SARS-CoV-2 positive by RT-PCR in nasal swab for 127 days PSO.

**Patient #5**

Patient #5 is a 48-year old patient of Turkish descendance and was diagnosed with Good’s syndrome during COVID-19. Seven days after symptom onset, patient #5 was admitted to hospital due to respiratory insufficiency. Mechanical ventilation was started on day 20 PSO. After unsuccessful treatment with dexamethasone, immunoglobulins and different antibiotics patient #5 was transferred to our center. He received 8g of Casirivimab/Imdevimab and ECMO treatment was initiated on day 30 PSO. Serological and virological follow-up showed detectable SARS-CoV-2-specific IgG but no specific IgA and viral clearance of SARS-CoV-2 in peripheral blood within 14 days post treatment. Patient #5 deceased 47 days after disease onset despite ECMO due septic shock.
